# Association of adverse drug reaction to anti-tuberculosis medication with quality of life in patients in a tertiary referral hospital

**DOI:** 10.1590/0037-8682-0207-2019

**Published:** 2019-12-20

**Authors:** Ronise Malaquias Carlos Valadares, Wânia da Silva Carvalho, Silvana Spíndola de Miranda

**Affiliations:** 1 Universidade Federal de Minas Gerais, Faculdade de Medicina, Programa de Pós-Graduação em Ciências Aplicadas à Saúde do Adulto, Belo Horizonte, MG, Brasil.; 2 Fundação Hospitalar do Estado de Minas Gerais, Hospital Júlia Kubitschek. Belo Horizonte, MG, Brasil.; 3 Universidade Federal de Minas Gerais, Faculdade de Farmácia, Departamento de Farmácia Social, Programa de Pós-Graduação em Medicamentos e Assistência Farmacêutica, Belo Horizonte, MG, Brasil.

**Keywords:** Tuberculosis, Anti-tuberculosis agents, Adverse reactions, Quality of life

## Abstract

**INTRODUCTION:**

Adverse drug reactions can develop when using anti-tuberculosis medication, and the effects of the drugs can also significantly hinder the treatment of patients.

**METHODS:**

A cross-sectional survey was conducted in 73 patients using two standardized questionnaires and the World Health Organization Quality of Life-Bref.

**RESULTS:**

All patients reported the presence of adverse drug reactions, 71.6% of which are minor and 28.3% both major and minor. The global quality of life analysis showed that patients with tuberculosis have a good average (67.3%).

**CONCLUSIONS:**

There is an association between quality of life and adverse drug reaction, educational level, and vulnerability.

Adverse drug reactions (ADRs) can develop when using anti-tuberculosis (antiTB) medication. In this case, the ADRs are classified as minor reactions, which do not usually result in the immediate modification of the standardized regimen, and major reactions, which normally result in an alteration or even discontinuation of the treatment regimen (3% to 8% of cases)^1^.

In patients receiving tuberculosis (TB) treatment, the main determinants of ADR are related to administration of drugs, dose and timing, age, nutritional status, alcoholism, liver function, renal function, and co-infection with the human immunodeficiency virus (HIV)^1^ ^,^ ^2^.

It is important to emphasize that TB and its consequences, including ADR, affect not only the physical and organic scope of patients but also the psycho-affective and social fields, directly interfering with the quality of life (QOL).

This study aimed to evaluate the antiTB ADRs in patients hospitalized at a tertiary referral hospital in Belo Horizonte, Minas Gerais (MG) and analyze its association with QOL.

The study was approved by the Research Ethics Committee of the Federal University of Minas Gerais and Minas Gerais State Hospital Foundation (FHEMIG).

This was a cross-sectional study conducted from March to December 2015 in adult patients with sensitive or resistant *Mycobacterium tuberculosis* at any stage of treatment (new cases, relapses, and re-admission after cessation). The patients were hospitalized at Hospital Júlia Kubitschek of the FHEMIG, in Belo Horizonte, MG, and a tertiary referral hospital for special cases with priorities related to TB.

Patient selection was not probabilistic and performed by means of evaluation of the inclusion criteria in all patients admitted during the research period. The inclusion criteria were as follows: age >18 years, full autonomy, diagnosis of TB, bacteriologically confirmed pulmonary TB, suitable physical and psychological conditions to participate in the study, antiTB medication use, and hospitalization at any stage of treatment. The exclusion criteria were as follows: other pulmonary diseases, non-tuberculous mycobacteria, and refusal to participate in the study or sign the informed consent form.

The independent variables evaluated were sociodemographic, clinical, and behavioral characteristics, pharmacological profile of antiTB medication (first-line regimen or others), use of symptomatic drugs, and ADR, and the dependent variable was QOL. The sociodemographic characteristics were as follows: sex, age, race/ethnicity, educational level, and vulnerability (prisoners, homeless population, healthcare professional, or immigrant). 

The clinical characteristics were as follows: type of entry (new case, relapse, and re-admission after cessation), type of TB (pulmonary or extrapulmonary), HIV serology (negative or positive), associated disorders (alcoholism, smoking, systemic arterial hypertension, diabetes mellitus, or other comorbidities), and sensitivity profile to drugs (sensitive or resistant). The length of exposure to the medications indicates the total number of days elapsed between the starting date of the use of medications and the date of the interview. 

The ADRs were classified into major, minor, and both^1^. The behavioral characteristics evaluated were alcoholism and smoking^3^ ^,^ ^4^.

Data from the independent variables were obtained through a patient interview conducted using two standardized questionnaires^3^ ^,^ ^4^. The antiTB pharmacotherapeutic profile was evaluated by direct prescription consultation.

The QOL assessment was conducted using the World Health Organization Quality of Life-Bref (WHOQOL-Bref)^5^. The QOL calculations were performed in an automated manner using the Microsoft Excel software following the syntax proposed by the WHOQOL Group. The univariate analysis of association included the global QOL; sociodemographic, behavioral, and clinical variables; ADRs; and pharmacological profile, and cases with a P-value <0.20 were included in the multiple linear regression analysis whose result considered the level of significance as a P-value <0.05. 

The sample consisted of 73 individuals with the following characteristics: male sex (54/73, 73.9%), age (>50 years, 49/73, 67.1%), literate (62/73, 84.9%), non-white (64/73, 87.7%), new case of TB (37/73, 50.7%), pulmonary TB (100%), HIV negative (72/73, 98.6%), alcoholism and smoking (35/73, 47.9%, and 38/73, 52.1%, respectively), and sensible susceptibility of the strains (51/73, 69.9%).

The length of exposure to antiTB medications was >3 days in 90.4% (66/73) of the patients, with a median of 8 days and interquartile range of 12.5. The reason for hospitalization of the majority of patients was a general state that did not allow treatment in the outpatient clinic (43/73, 58.9%). In 6 (8.2%) patients, the reason was intolerance to anti-TB medications. There were 24 patients (32.8%) with social vulnerability (absence of fixed residence) with the greatest possibility of cessation, relapse, failure, or multi-resistance.

The first-line regimen was used by 51 (69.8%) patients, and 49 (96.0%) were in the intensive phase (rifampicin (R) 150 mg + isoniazid (H) - 75 mg + pyrazinamide (Z) 400 mg + ethambutol (E) 275 mg) and 2 (3.9%) were in the maintenance phase (R150 + H75). Other patients (22/73, 30.1%) were using a special scheme.

All patients (100%) reported having had one or more ADRs to anti-TB medications, regardless of the regimen used. Regarding ADR classification, 56 patients (76.7%) reported only minor ADR, and 17 (23.2%) reported minor and major ADRs. No patient reported the presence of only major ADR. The major ADRs reported were hepatitis, psychosis, and rashes.

Table 1 presents the ADRs to antiTB medications that were self-reported by patients. Alteration of the treatment regimen occurred in 3 patients (17.6%) with minor and major ADRs. The use of symptomatic drugs for ADR was frequent among patients, with analgesics being the most common therapeutic class (66/73, 90.4%), followed by antiemetics (62/73, 84.9%). The other drugs were antihistamines (4/73, 5.5%), anti-inflammatories (3/73, 4.1%), anxiolytics (15/73, 20.5%), and vitamin B6 (31/73, 42.5%).

**TABLE 1:** Adverse drug reactions to anti-tuberculosis medications self-reported by patients (n=73).

Adverse drug reactions		Patients with ADR	
		n	%
Gastrointestinal	Increased appetite	36	49.3
	Nausea	28	38.3
	Anorexia	21	28.8
	Diarrhea	20	27.4
	Epigastralgia	18	24.7
	Constipation	15	20.5
	Heartburn	12	16.4
	Hepatitis	2	2.7
Dermatological	Pruritus	24	32.9
	Exfoliative dermatitis	16	21.9
	Rash	9	12.3
	Acneiform spots	2	2.7
Osteoarticular	Arthralgia	21	28.8
	Arthritis	6	8.2
Psychiatric	Emotionalism	34	46.6
	Anxiety	30	41.1
	Alterations in memory and concentration	18	24.6
	Psychosis	12	16.4
	Aggression	9	12.3
	Euphoria	5	6.8
Neurological	Dry mouth	41	56.2
	Postural hypotension	34	46.6
	Insomnia	27	37.0
	Drowsiness	18	24.6
	Vestibulocochlear toxicity	3	4.1
Hematological	Anemia	8	11.0
Renal	Dysuria	41	56.2
	Difficulty in urination	5	6.8
Other effects	Red urine	50	68.5
	Red feces	26	35.6
	Alteration in the visual field	26	35.6
	Fever	10	13.7
	Flu	3	4.1
	Oligo- or amenorrhea	3	4.1
	Alteration in color vision	1	1.4
	Asthma	1	1.4

The results of the WHOQOL-Bref questionnaire presented in Table 2 show that the average global QOL was 67.3%. The averages in the psychological and environmental domains were the largest. The correlation between QOL and its domains was significant in all cases; however, it is stronger in the psychological domain (Table 2). 

**TABLE 2:** Average, percentage, pattern deviation, and correlation coefficient of Spearman quality of life (QOL) with physical, psychological, social relations, and environmental domains (n=73).

Area	Average	%	Pattern deviation	Global coefficient	P-value
Global	13.5	67.3	1.50	1.0	
Physical	12.2	61.2	1.96	0.664	<0.001
Psychological	14.5	72.7	2.22	0.803	<0,001
Social relations	11.3	56.4	2.92	0.390	<0.001
Environmental	14.3	71.3	1.82	0.642	<0.001

The univariate analysis of the association between global QOL and independent variables (ADR, sociodemographic, behavioral, and clinical characteristics, and antiTB medications) was significant for the type of ADR (p=0.016), educational level (p=0.022) (p=0.076), vulnerability (p=0.109), and antiTB medication (p=0.092) (p<0.2) (Table 3).

The multivariate model found an association between the global QOL and minor ADRs, and minor and major ADRs, homelessness, and elementary and higher education, considering a P-value <0.05 (Table 3).

Patients with minor and major ADRs had a QOL score that decreased by 1.04 points compared to individuals who reported only minor ADR. Additionally, patients belonging to the homeless population had a QOL score that decreased by 1.63 points compared to that in prisoners, and the illiterate population had a QOL score that decreased by 2.18 and 0.84 points compared to the higher and elementary education populations, respectively (Table 3). 

**TABLE 3:** Inferential analysis of quality of life with sociodemographic, behavioral, and clinical variables and adverse drug reactions to anti-tuberculosis medications (n=73).

Variable	Univariate		Multivariate	
	P-value	Confidence Interval (95%)	Coefficient	P-value
Sex	0.643	(-0.990-0.0615)		
Female				
Male				
Educational level				
Illiterate				
Primary	0.022	(0.276-3.388)	0.848	0.047
Secondary	0.060	(-0.070-3.419)		
Higher	0.013	(0.580-4.805)	2.185	0.008
Unknown				
Age, years	0.076	(-1.398-0.071)		
18-40				
>40				
Race/ethnicity				
White				
Black	0.806	(-1.056-1.354)		
Olive-skinned	0.540	(-0.766-1.452)		
Special population				
Prisoner				
Homeless population	0.109	(-3.600-0.370)		
No	0.623	(-2.163-1.304)	-1.634	0.006
Input type				
New case				
Relapse	0.794	(-0.805-1.048)		
Re-admitted after cessation	0.645	(-1.019-0.635)		
Length of exposure	0.696	-(0.004-0.005)		
Associated disorders				
Alcoholism	0.939	(-0.679-0.733)		
Smoking	0.864	(-0.767-0645)		
Sensibility profile	0.092	(-0.108-1.399)		
Sensitive				
Resistant				
Type of ADR	0.016	(-1.793-0.192)	-1.040	0.012

Few studies have been conducted to evaluate the association between QOL and ADR to antiTB medications in patients admitted to a tertiary referral hospital and those with other diseases^6^. This study has shown the association between QOL and ADR and other characteristics such as educational level and vulnerability. Patients who have had minor and major ADRs (clinically more severe) may have increased morbidity and longer treatment duration, probably due to major ADRs; therefore, having minor and major ADRs is worse than having minor ADR in the global QOL score. 

The lower overall QOL score in the homeless population compared to that in prisoners is probably due to the living conditions of these individuals who do not have adequate shelter or food, unlike those who live in a physical environment that provides them with healthcare and food even if they are deprived of living with family and friends. 

Regarding the illiterate population, the global QOL is impaired due to limitations related to the ability to obtain health-related information and education, as well as impairment of self-esteem. This is corroborated by another study that found a positive relationship between QOL and education and suggests that education may be a way to improve the QOL of patients with TB^7^.

In this study, the global QOL analysis considered that patients with TB presented good QOL (67.3%). The highest averages of QOL were observed in the psychological and environmental domains, probably due to the specialized care by the multiprofessional team and the physical space of HJK’s phthisiology ward, which has adequate facilities.

Some authors found diverse results regarding the influence of scores in the two domains, presenting the physical and social domains as the most relevant since these were associated with the clinical conditions and outpatient treatment regimen^8^.

Another study found the best QOL average in the social relations domain, which is justified by the culture and religion of the studied population that promotes social relations^7^.

The worst score was related to the social relations domain, probably due to the patient’s withdrawal from their family and friends, and the lowest average was from sexual activity domain. This result is consistent with another study that showed bias toward patients with TB and those closest to them, which is a contributing factor to their isolation from society, mainly due to the stigma and fear of transmission of the disease^9^. 

As the data in this study was collected by means of a standardized questionnaire (primary data), the most frequently observed ADRs were red urine, dysuria, and dry mouth, which were different from those of other studies that reported that the most frequently observed ADRs were joint pain and skin edema and irritation^10^. Probably, it may be due to the data obtained from medical records, where less severe effects may have been disregarded^10^.

Manifestations related to gastrointestinal, neurological, and other effects and psychiatric systems were the most frequent ADRs. Moreover, another study found a higher frequency of ADR in the musculoskeletal and visual systems^11^.

According to the Ministry of Health of Brazil, whose information is based on secondary sources such as health services and medical record notifications (instruments heavily influenced by underreporting), the prevalence rate of major ADRs that call for an alteration in the treatment regimen was 3% to 8%^1^. However, in our study, we found alterations in 17.6% (3/17) of patients with major and minor ADRs, a difference most likely due to the collection of primary information (applied questionnaire).

It was noted that the majority of patients was male, reported a primary and secondary educational level, was aged >40 years, had black race, and resided in the capital, which were similar to those in other studies in which TB affects individuals with these sociodemographic characteristics^8^ ^,^ ^12^ ^,^ ^13^. Regarding clinical characteristics, there was a predominance of new cases with the use of first-line regimen and pulmonary involvement with drug-sensitive strains of *M. tuberculosis*, which is in accordance with the epidemiological data of the Ministry of Health^14^. The special scheme is verified to be used in the management of patient cases in retreatment, failure, multi-resistance, or drug intolerance^2^ ^,^ ^15^. In relation to behavioral characteristics, the percentages of patients with alcoholism and those who smoke were similar, which were also described by other authors, considering that the same patient could present with both comorbidities^12^. This study has some limitations. It is a cross-sectional study and did not evaluate the cognition of patients (mini mental). Therefore, there is an association between QOL and ADR, educational level, and vulnerability.
